# *N*-[(5-Chloro-3-methyl-1-phenyl-1*H*-pyrazol-4-yl)carbon­yl]-*N*′-(4-hydroxy­phen­yl)thio­urea

**DOI:** 10.1107/S1600536808019417

**Published:** 2008-07-05

**Authors:** Haitang Du, Haijun Du, Ying An, Shengnan Li

**Affiliations:** a Department of Biology and Environment Technology, Guiyang College, Guiyang 550005, People’s Republic of China; b School of Chemistry and Environment Science, Guizhou University for Nationalities, Guiyang 550025, People’s Republic of China; c Department of Chemistry, College of Science, Tianjin University, Tianjin 300072, People’s Republic of China

## Abstract

In the title compound, C_18_H_15_ClN_4_O_2_S, the pyrazole ring makes dihedral angles of 67.4 (1) and 12.5 (1)° with the phenyl and 4-hydroxy­phenyl groups, respectively; the two benzene rings are twisted by 60.1 (1)° with respect to each other. The thio­urea NH groups are involved in N—H⋯O and N—H⋯Cl intra­molecular hydrogen bonds. A hydrogen bond between the phenolic OH group and the pyrazole N atom connects mol­ecules into a one-dimensional polymeric structure.

## Related literature

For related literature, see: Du *et al.* (2007 ▶); Saeed & Flörke (2007 ▶); Wang *et al.* (2007 ▶).
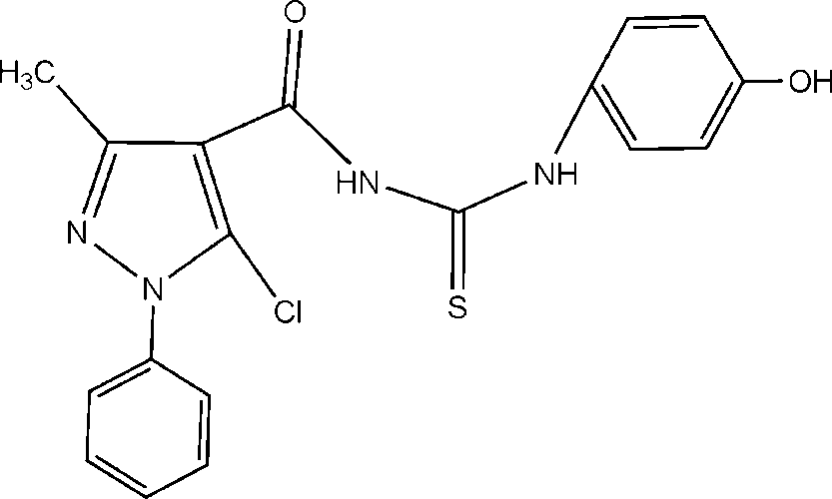


## Experimental

### 

#### Crystal data

C_18_H_15_ClN_4_O_2_S
*M* *_r_* = 386.85
Triclinic, 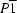

*a* = 8.572 (2) Å
*b* = 10.429 (2) Å
*c* = 11.170 (2) Å
α = 99.936 (4)°
β = 105.817 (4)°
γ = 106.042 (4)°
*V* = 889.5 (3) Å^3^
*Z* = 2
Mo *K*α radiation
μ = 0.35 mm^−1^
*T* = 294 (2) K
0.26 × 0.24 × 0.20 mm

#### Data collection

Bruker SMART 1K CCD diffractometer
Absorption correction: multi-scan (*SADABS*; Sheldrick, 1996 ▶) *T*_min_ = 0.914, *T*_max_ = 0.933
4615 measured reflections
3118 independent reflections
2160 reflections with *I* > 2σ(*I*)
*R*_int_ = 0.025

#### Refinement

*R*[*F* ^2^ > 2σ(*F* ^2^)] = 0.045
*wR*(*F* ^2^) = 0.122
*S* = 1.04
3118 reflections
245 parameters
2 restraints
H atoms treated by a mixture of independent and constrained refinement
Δρ_max_ = 0.20 e Å^−3^
Δρ_min_ = −0.35 e Å^−3^

### 

Data collection: *SMART* (Bruker, 1997 ▶); cell refinement: *SAINT* (Bruker, 1997 ▶); data reduction: *SAINT*; program(s) used to solve structure: *SHELXS97* (Sheldrick, 2008 ▶); program(s) used to refine structure: *SHELXL97* (Sheldrick, 2008 ▶); molecular graphics: *SHELXTL* (Sheldrick, 2008 ▶); software used to prepare material for publication: *SHELXTL*.

## Supplementary Material

Crystal structure: contains datablocks I, global. DOI: 10.1107/S1600536808019417/gk2153sup1.cif

Structure factors: contains datablocks I. DOI: 10.1107/S1600536808019417/gk2153Isup2.hkl

Additional supplementary materials:  crystallographic information; 3D view; checkCIF report

## Figures and Tables

**Table 1:** Hydrogen-bond geometry (Å, °)

*D*—H⋯*A*	*D*—H	H⋯*A*	*D*⋯*A*	*D*—H⋯*A*
O2—H2⋯N2^i^	0.82	2.15	2.938 (3)	162
N3—H3*A*⋯Cl1	0.891 (10)	2.422 (19)	3.168 (2)	141 (2)
N4—H4*A*⋯O1	0.901 (10)	1.92 (2)	2.661 (3)	139 (2)

Symmetry code: (i) 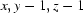.

**Fig. 1.**
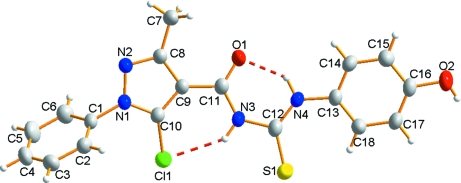
The molecular structure of the title compound with the atom numbering scheme, showing displacement ellipsoids at the 50% probability level.

**Fig. 2.**
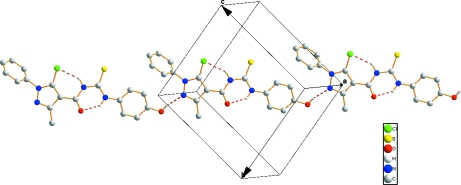
The polymeric structure via O-H···N hydrogen bonds. Hydrogen bonds are shown with dashed lines.

## supplementary crystallographic information

### Comment

The title compound is similar to the previously reported N-(5-chloro-3-methyl-1-phenylpyrazole-4-ylcarbonyl)-N'- (4-methphenyl)thiourea (Du *et al.*, 2007). The molecular structure of the title compound and the atom-numbering scheme are shown in Fig.1. The pyrazole ring makes dihedral angles of 67.4 (1) and 12.5 (1)°, with the C1—C6 and C13—C18 rings, respectively. These two six-membered rings are twisted by 60.1 (1)° with respect to each other. This geometry is stabilized by intramolecular N4-H4A ···O1 and N3-H3A···Cl hydrogen bonds (Fig.1, Table 1). In the crystal structure, molecules are linked by intermolecular N-H···O hydrogen bonds to form a one-dimensional polymeric structure (Fig.2). All bond lengths and angles are in the normal range (Du *et al.*, 2007; Saeed & Flörke, 2007; Wang *et al.*, 2007).

### Experimental

Powdered ammonium thiocyanate (15 mmol), 5-chloro-3-methyl-1-phenyl-pyrazole-4-carbonyl chloride (10 mmol), PEG-400 (0.5 mL) and acetone (25 mL) were placed in a dried round-bottom flask and stirred at room temperature for 1 h, then 4-aminophenol (9.5 mmol) was added, and the mixture was stirred for 5 h. The mixture was poured into water (20 mL). The resulting solid was filtered, dried and recrystallized from *N*,*N*-dimethylformamide/ethanol to give the title compound. Single crystals were obtained by slow evaporation of a solution in *N*,*N*-dimethylformamide/ethanol (1:1, *v*/*v*).

### Refinement

H atoms bonded to N atoms were located in a difference Fourier map and refined with distance restraints (N—H = 0.89 Å) and with *U*_iso_(H) = 1.2*U*_eq_(N). Other H atoms were positioned geometrically and refined using a riding model, with C—H = 0.93–0.96 Å and O–H = 0.82 Å;, *U*_iso_(H) = *xU*_eq_(carrier atom) where *x* = 1.5 for methyl groups and 1.2 for the remaining atoms.

### Crystal data

**Table:**

C_18_H_15_ClN_4_O_2_S	*Z* = 2
*M**_r_* = 386.85	*F*_000_ = 400
Triclinic, *P*1	*D*_x_ = 1.444 Mg m^−^^3^
Hall symbol: -P 1	Melting point: 456 K
*a* = 8.572 (2) Å	Mo *K*α radiation λ = 0.71073 Å
*b* = 10.429 (2) Å	Cell parameters from 1626 reflections
*c* = 11.170 (2) Å	θ = 2.6–25.0º
α = 99.936 (4)º	µ = 0.35 mm^−^^1^
β = 105.817 (4)º	*T* = 294 (2) K
γ = 106.042 (4)º	Prism, colorless
*V* = 889.5 (3) Å^3^	0.26 × 0.24 × 0.20 mm

### Data collection

**Table:**

Bruker SMART 1K CCD diffractometer	3118 independent reflections
Radiation source: fine-focus sealed tube	2160 reflections with *I* > 2σ(*I*)
Monochromator: graphite	*R*_int_ = 0.025
*T* = 294(2) K	θ_max_ = 25.0º
φ and ω scans	θ_min_ = 2.0º
Absorption correction: multi-scan(SADABS; Sheldrick, 1996)	*h* = −10→7
*T*_min_ = 0.914, *T*_max_ = 0.933	*k* = −12→12
4615 measured reflections	*l* = −10→13

### Refinement

**Table:**

Refinement on *F*^2^	Secondary atom site location: difference Fourier map
Least-squares matrix: full	Hydrogen site location: inferred from neighbouring sites
*R*[*F*^2^ > 2σ(*F*^2^)] = 0.045	H atoms treated by a mixture of independent and constrained refinement
*wR*(*F*^2^) = 0.123	*w* = 1/[σ^2^(*F*_o_^2^) + (0.0601*P*)^2^ + 0.0911*P*] where *P* = (*F*_o_^2^ + 2*F*_c_^2^)/3
*S* = 1.04	(Δ/σ)_max_ = 0.001
3118 reflections	Δρ_max_ = 0.20 e Å^−^^3^
245 parameters	Δρ_min_ = −0.35 e Å^−^^3^
2 restraints	Extinction correction: none
Primary atom site location: structure-invariant direct methods	

### Special details

**Table:**

Geometry. All e.s.d.'s (except the e.s.d. in the dihedral angle between two l.s. planes) are estimated using the full covariance matrix. The cell e.s.d.'s are taken into account individually in the estimation of e.s.d.'s in distances, angles and torsion angles; correlations between e.s.d.'s in cell parameters are only used when they are defined by crystal symmetry. An approximate (isotropic) treatment of cell e.s.d.'s is used for estimating e.s.d.'s involving l.s. planes.
Refinement. Refinement of *F*^2^ against ALL reflections. The weighted *R*-factor *wR* and goodness of fit *S* are based on *F*^2^, conventional *R*-factors *R* are based on *F*, with *F* set to zero for negative *F*^2^. The threshold expression of *F*^2^ > σ(*F*^2^) is used only for calculating *R*-factors(gt) *etc*. and is not relevant to the choice of reflections for refinement. *R*-factors based on *F*^2^ are statistically about twice as large as those based on *F*, and *R*- factors based on ALL data will be even larger.

### Fractional atomic coordinates and isotropic or equivalent isotropic displacement parameters (Å^2^)

**Table:**

	*x*	*y*	*z*	*U*_iso_*/*U*_eq_	
Cl1	0.12809 (11)	0.51284 (7)	0.87995 (7)	0.0638 (3)	
S1	0.24176 (14)	0.24348 (8)	0.57180 (8)	0.0743 (3)	
O1	0.2267 (3)	0.66532 (18)	0.52727 (17)	0.0588 (6)	
O2	0.4029 (3)	0.19520 (19)	0.00036 (17)	0.0553 (5)	
H2	0.3689	0.1104	−0.0168	0.083*	
N1	0.1986 (3)	0.78380 (19)	0.93012 (18)	0.0391 (5)	
N2	0.2336 (3)	0.8960 (2)	0.88065 (18)	0.0419 (5)	
N3	0.2115 (3)	0.4873 (2)	0.6192 (2)	0.0462 (6)	
N4	0.2649 (3)	0.4289 (2)	0.43005 (19)	0.0441 (5)	
C1	0.1829 (3)	0.7990 (2)	1.0561 (2)	0.0380 (6)	
C2	0.3043 (4)	0.7804 (3)	1.1543 (2)	0.0464 (7)	
H2A	0.3950	0.7556	1.1394	0.056*	
C3	0.2892 (4)	0.7994 (3)	1.2762 (3)	0.0533 (7)	
H3	0.3708	0.7883	1.3442	0.064*	
C4	0.1535 (4)	0.8345 (3)	1.2966 (3)	0.0582 (8)	
H4	0.1431	0.8462	1.3782	0.070*	
C5	0.0335 (4)	0.8525 (3)	1.1974 (3)	0.0622 (8)	
H5	−0.0579	0.8763	1.2119	0.075*	
C6	0.0477 (4)	0.8354 (3)	1.0758 (3)	0.0506 (7)	
H6	−0.0329	0.8483	1.0084	0.061*	
C7	0.2683 (4)	0.9426 (3)	0.6808 (2)	0.0564 (8)	
H7A	0.2970	1.0362	0.7298	0.085*	
H7B	0.1667	0.9190	0.6068	0.085*	
H7C	0.3624	0.9343	0.6529	0.085*	
C8	0.2354 (3)	0.8466 (2)	0.7635 (2)	0.0384 (6)	
C9	0.2041 (3)	0.7020 (2)	0.7349 (2)	0.0362 (6)	
C10	0.1803 (3)	0.6678 (2)	0.8446 (2)	0.0391 (6)	
C11	0.2136 (3)	0.6184 (2)	0.6186 (2)	0.0388 (6)	
C12	0.2401 (3)	0.3889 (3)	0.5323 (2)	0.0429 (6)	
C13	0.3013 (3)	0.3615 (2)	0.3244 (2)	0.0395 (6)	
C14	0.3412 (4)	0.4386 (3)	0.2413 (2)	0.0490 (7)	
H14	0.3439	0.5298	0.2574	0.059*	
C15	0.3771 (4)	0.3835 (3)	0.1354 (3)	0.0522 (7)	
H15	0.4046	0.4376	0.0810	0.063*	
C16	0.3726 (3)	0.2478 (3)	0.1094 (2)	0.0415 (6)	
C17	0.3379 (4)	0.1723 (3)	0.1931 (2)	0.0504 (7)	
H17	0.3389	0.0821	0.1780	0.061*	
C18	0.3012 (4)	0.2268 (3)	0.2999 (3)	0.0535 (8)	
H18	0.2765	0.1730	0.3551	0.064*	
H3A	0.205 (4)	0.459 (3)	0.6890 (18)	0.066 (9)*	
H4A	0.260 (4)	0.5134 (15)	0.426 (3)	0.064 (9)*	

### Atomic displacement parameters (Å^2^)

**Table:**

	*U*^11^	*U*^22^	*U*^33^	*U*^12^	*U*^13^	*U*^23^
Cl1	0.1074 (7)	0.0351 (4)	0.0593 (5)	0.0189 (4)	0.0486 (4)	0.0147 (3)
S1	0.1377 (9)	0.0495 (5)	0.0739 (6)	0.0502 (5)	0.0681 (6)	0.0290 (4)
O1	0.1072 (17)	0.0502 (11)	0.0377 (10)	0.0421 (12)	0.0355 (11)	0.0157 (9)
O2	0.0702 (14)	0.0514 (11)	0.0490 (11)	0.0212 (11)	0.0335 (10)	0.0029 (9)
N1	0.0546 (14)	0.0326 (11)	0.0314 (11)	0.0138 (10)	0.0196 (10)	0.0054 (9)
N2	0.0624 (15)	0.0350 (11)	0.0338 (11)	0.0189 (10)	0.0223 (10)	0.0096 (9)
N3	0.0740 (17)	0.0357 (12)	0.0405 (13)	0.0245 (11)	0.0317 (12)	0.0098 (10)
N4	0.0695 (16)	0.0334 (12)	0.0375 (12)	0.0248 (11)	0.0240 (11)	0.0082 (10)
C1	0.0495 (17)	0.0319 (13)	0.0332 (13)	0.0127 (12)	0.0187 (12)	0.0046 (10)
C2	0.0603 (19)	0.0426 (15)	0.0458 (16)	0.0244 (14)	0.0247 (14)	0.0135 (12)
C3	0.076 (2)	0.0499 (16)	0.0380 (15)	0.0244 (15)	0.0209 (14)	0.0154 (12)
C4	0.085 (2)	0.0532 (17)	0.0442 (17)	0.0223 (17)	0.0366 (17)	0.0110 (14)
C5	0.068 (2)	0.075 (2)	0.0599 (19)	0.0313 (17)	0.0414 (17)	0.0162 (16)
C6	0.0510 (18)	0.0583 (18)	0.0478 (16)	0.0229 (14)	0.0205 (13)	0.0142 (13)
C7	0.095 (2)	0.0413 (15)	0.0418 (15)	0.0289 (16)	0.0298 (16)	0.0146 (12)
C8	0.0502 (16)	0.0359 (13)	0.0311 (13)	0.0182 (12)	0.0142 (11)	0.0080 (11)
C9	0.0457 (16)	0.0332 (13)	0.0305 (12)	0.0154 (11)	0.0135 (11)	0.0062 (10)
C10	0.0493 (16)	0.0330 (13)	0.0366 (14)	0.0150 (12)	0.0175 (12)	0.0067 (11)
C11	0.0460 (16)	0.0373 (14)	0.0324 (13)	0.0164 (12)	0.0124 (11)	0.0054 (11)
C12	0.0509 (17)	0.0373 (14)	0.0398 (14)	0.0156 (12)	0.0183 (12)	0.0032 (11)
C13	0.0491 (16)	0.0352 (13)	0.0339 (13)	0.0164 (12)	0.0154 (12)	0.0028 (11)
C14	0.071 (2)	0.0328 (13)	0.0509 (16)	0.0221 (14)	0.0291 (15)	0.0094 (12)
C15	0.071 (2)	0.0446 (16)	0.0491 (16)	0.0214 (14)	0.0310 (15)	0.0135 (13)
C16	0.0436 (16)	0.0398 (14)	0.0379 (14)	0.0141 (12)	0.0155 (12)	−0.0001 (11)
C17	0.076 (2)	0.0356 (14)	0.0448 (15)	0.0237 (14)	0.0276 (14)	0.0049 (12)
C18	0.088 (2)	0.0389 (15)	0.0445 (16)	0.0248 (15)	0.0351 (15)	0.0126 (12)

### Geometric parameters (Å, °)

**Table:**

Cl1—C10	1.698 (2)	C4—H4	0.9300
S1—C12	1.654 (3)	C5—C6	1.382 (4)
O1—C11	1.224 (3)	C5—H5	0.9300
O2—C16	1.369 (3)	C6—H6	0.9300
O2—H2	0.8200	C7—C8	1.496 (3)
N1—C10	1.347 (3)	C7—H7A	0.9600
N1—N2	1.373 (3)	C7—H7B	0.9600
N1—C1	1.436 (3)	C7—H7C	0.9600
N2—C8	1.327 (3)	C8—C9	1.419 (3)
N3—C11	1.363 (3)	C9—C10	1.384 (3)
N3—C12	1.407 (3)	C9—C11	1.470 (3)
N3—H3A	0.891 (10)	C13—C14	1.380 (3)
N4—C12	1.331 (3)	C13—C18	1.384 (3)
N4—C13	1.421 (3)	C14—C15	1.374 (3)
N4—H4A	0.901 (10)	C14—H14	0.9300
C1—C6	1.374 (4)	C15—C16	1.382 (3)
C1—C2	1.374 (4)	C15—H15	0.9300
C2—C3	1.389 (4)	C16—C17	1.363 (4)
C2—H2A	0.9300	C17—C18	1.384 (3)
C3—C4	1.375 (4)	C17—H17	0.9300
C3—H3	0.9300	C18—H18	0.9300
C4—C5	1.371 (4)		
			
C16—O2—H2	109.5	H7B—C7—H7C	109.5
C10—N1—N2	110.66 (18)	N2—C8—C9	111.7 (2)
C10—N1—C1	128.6 (2)	N2—C8—C7	119.6 (2)
N2—N1—C1	120.68 (17)	C9—C8—C7	128.7 (2)
C8—N2—N1	105.50 (18)	C10—C9—C8	103.6 (2)
C11—N3—C12	130.5 (2)	C10—C9—C11	130.9 (2)
C11—N3—H3A	118.6 (19)	C8—C9—C11	125.2 (2)
C12—N3—H3A	110.4 (19)	N1—C10—C9	108.6 (2)
C12—N4—C13	130.9 (2)	N1—C10—Cl1	120.04 (18)
C12—N4—H4A	115.9 (18)	C9—C10—Cl1	131.33 (19)
C13—N4—H4A	113.2 (18)	O1—C11—N3	121.8 (2)
C6—C1—C2	121.7 (2)	O1—C11—C9	121.6 (2)
C6—C1—N1	118.6 (2)	N3—C11—C9	116.6 (2)
C2—C1—N1	119.7 (2)	N4—C12—N3	114.1 (2)
C1—C2—C3	118.7 (3)	N4—C12—S1	129.42 (19)
C1—C2—H2A	120.6	N3—C12—S1	116.46 (19)
C3—C2—H2A	120.6	C14—C13—C18	118.3 (2)
C4—C3—C2	120.1 (3)	C14—C13—N4	116.5 (2)
C4—C3—H3	120.0	C18—C13—N4	125.3 (2)
C2—C3—H3	120.0	C15—C14—C13	121.4 (2)
C5—C4—C3	120.4 (3)	C15—C14—H14	119.3
C5—C4—H4	119.8	C13—C14—H14	119.3
C3—C4—H4	119.8	C14—C15—C16	120.1 (2)
C4—C5—C6	120.3 (3)	C14—C15—H15	119.9
C4—C5—H5	119.9	C16—C15—H15	119.9
C6—C5—H5	119.9	C17—C16—O2	122.9 (2)
C1—C6—C5	118.9 (3)	C17—C16—C15	118.7 (2)
C1—C6—H6	120.5	O2—C16—C15	118.3 (2)
C5—C6—H6	120.5	C16—C17—C18	121.6 (2)
C8—C7—H7A	109.5	C16—C17—H17	119.2
C8—C7—H7B	109.5	C18—C17—H17	119.2
H7A—C7—H7B	109.5	C13—C18—C17	119.9 (2)
C8—C7—H7C	109.5	C13—C18—H18	120.1
H7A—C7—H7C	109.5	C17—C18—H18	120.1
			
C10—N1—N2—C8	0.3 (3)	C11—C9—C10—N1	173.8 (3)
C1—N1—N2—C8	−178.5 (2)	C8—C9—C10—Cl1	176.7 (2)
C10—N1—C1—C6	−112.6 (3)	C11—C9—C10—Cl1	−8.8 (4)
N2—N1—C1—C6	66.0 (3)	C12—N3—C11—O1	7.4 (4)
C10—N1—C1—C2	69.0 (3)	C12—N3—C11—C9	−171.0 (3)
N2—N1—C1—C2	−112.5 (3)	C10—C9—C11—O1	176.2 (3)
C6—C1—C2—C3	−0.1 (4)	C8—C9—C11—O1	−10.4 (4)
N1—C1—C2—C3	178.2 (2)	C10—C9—C11—N3	−5.4 (4)
C1—C2—C3—C4	0.7 (4)	C8—C9—C11—N3	168.0 (2)
C2—C3—C4—C5	−0.7 (4)	C13—N4—C12—N3	178.3 (2)
C3—C4—C5—C6	0.0 (5)	C13—N4—C12—S1	−0.5 (4)
C2—C1—C6—C5	−0.5 (4)	C11—N3—C12—N4	−3.7 (4)
N1—C1—C6—C5	−178.9 (2)	C11—N3—C12—S1	175.3 (2)
C4—C5—C6—C1	0.6 (4)	C12—N4—C13—C14	−172.2 (3)
N1—N2—C8—C9	−0.8 (3)	C12—N4—C13—C18	7.1 (4)
N1—N2—C8—C7	179.3 (2)	C18—C13—C14—C15	1.2 (4)
N2—C8—C9—C10	1.0 (3)	N4—C13—C14—C15	−179.4 (3)
C7—C8—C9—C10	−179.2 (3)	C13—C14—C15—C16	0.5 (4)
N2—C8—C9—C11	−174.0 (2)	C14—C15—C16—C17	−2.2 (4)
C7—C8—C9—C11	5.9 (4)	C14—C15—C16—O2	177.6 (3)
N2—N1—C10—C9	0.3 (3)	O2—C16—C17—C18	−177.5 (3)
C1—N1—C10—C9	179.0 (2)	C15—C16—C17—C18	2.4 (4)
N2—N1—C10—Cl1	−177.47 (17)	C14—C13—C18—C17	−1.0 (4)
C1—N1—C10—Cl1	1.2 (4)	N4—C13—C18—C17	179.6 (3)
C8—C9—C10—N1	−0.7 (3)	C16—C17—C18—C13	−0.8 (4)

### Hydrogen-bond geometry (Å, °)

**Table:**

*D*—H···*A*	*D*—H	H···*A*	*D*···*A*	*D*—H···*A*
O2—H2···N2^i^	0.82	2.15	2.938 (3)	162
N3—H3A···Cl1	0.891 (10)	2.422 (19)	3.168 (2)	141 (2)
N4—H4A···O1	0.901 (10)	1.92 (2)	2.661 (3)	139 (2)

Symmetry codes: (i) *x*, *y*−1, *z*−1.
